# The emerging COVID-19 research: dynamic and regularly updated science maps and analyses

**DOI:** 10.1186/s12911-020-01321-9

**Published:** 2020-11-30

**Authors:** Njål Andersen, Jørgen G. Bramness, Ingunn Olea Lund

**Affiliations:** 1 Department of Leadership and Organizational Behaviour, BI Norwegian Business School, 0442 Oslo, Norway; 2 Norwegian Institute of Public Health, PO Box 222, 0213 Skøyen, Oslo, Norway; 3 UiT – The Arctic University of Norway, PO Box 6050, 9037 Langnes, Tromsø, Norway

**Keywords:** Bibliometrics, Science mapping, COVID-19, Keyword co-occurrence, Coupling analyses, Network graphs

## Abstract

**Background:**

The COVID-19 pandemic is a global public health emergency and experts emphasize the need for rapid and a high degree of communication and interaction between all parties, in order for critical research to be implemented. We introduce a resource (website) that provides bibliometric analysis showing the current content and structure of the published literature. As new research is published daily, the analysis is regularly updated to show the status as the research field develops and matures.

**Methods:**

Two bibliometric methods were employed, the first is a keyword co-occurrence analysis, based on published work available from PubMed. The second is a bibliometric coupling analysis, based on articles available through Scopus. The results are presented as clustered network graphs; available as interactive network graphs through the webpage.

**Results:**

For research as of March 23rd, keyword co-occurrence analysis showed that research was organized in 4 topic clusters: “Health and pandemic management”, “The disease and its pathophysiology”, “Clinical epidemiology of the disease” and “Treatment of the disease”. Coupling analyses resulted in 4 clusters on literature that relates to “Overview of the new virus”, “Clinical medicine”, “On the virus” and “Reproduction rate and spread”.

**Conclusion:**

We introduced a dynamic resource that will give a wide readership an overview of how the structure of the COVID-19 literature is developing. To illustrate what this can look like, we showed the structure as it stands three months after the virus was identified; the structure is likely to change as we progress to later stages of this pandemic.

## Background

On January 30th 2020, the World Health Organization (WHO) declared the COVID-19 outbreak a Public Health Emergency of International Concern and on March 11th categorized it as a pandemic. From its probable origin in China, it has now spread throughout the world. How the pandemic will progress and the associated consequences are still unknown. However, as of March 16th, several countries, ranging from large Asian countries like Korea to the small Nordic ones, have imposed strict nationwide sanctions previously unseen in peacetime. Measures include the closing of gyms and most cultural, sporting and leisure activities, conferences, concerts and other events are cancelled. Employees are required to work from home, and coupled with the closing of kindergartens, schools, colleges and universities who have switched to online teaching, many parents also have to engage in home schooling. As of March 28th the global death toll stands at 29,937 [1].

Governments and various funding bodies around the world are devoting large sums on research into COVID-19. World experts met at the World Health Organization’s headquarters in February to assess the current level of knowledge about COVID-19 and identify the most urgent research areas. They concluded with a call on the global research community to accelerate and fund priority research that can contribute to curtail the outbreak and prepare for future outbreaks [2]. The WHO identified the following main focus areas: identify origins and transmission, diagnosis, prevention and control, evaluation of therapeutics and vaccines and sharing of clinical research and data, optimal standard of care, supportive care practices. A final area was for relevant parties around the world to share, communicate and interact, so that critical research can be implemented [2]. The present study aims at supporting this, by giving an overview of current and emerging research and a way to identify relevant research.

There is a rapidly developing body of research on a broad range of topics related to COVID-19, with approximately 2000 new research items added to PubMed each week in the five months from April to August. Bibliometric science mapping and analysis is a quantitative approach to analyse, organize and review published research, based on manuscript metadata and bibliographic variables in each manuscript, and thus lends itself to rapidly analysing large bodies of work [3]. Traditional reviews, including traditional bibliometric reviews, show the state of research at a given point of time, and for slow moving research fields are valuable sources of scientific insight. However, with volumes of research being published as fast as during this pandemic, a traditional reviews format would quickly become obsolete. We therefore propose a novel variation to a bibliometric science mapping, where we introduce the aim, scope, method and preliminary results in this article, and provide the results on a dedicated website, updated regularly to include the emerging research, offering researchers a continuously  up-to-date overview of available research.

We provide analysis in two primary areas. The first is a keyword co-occurrence analysis, which provides an empirically grounded classification of concepts or taxonomy of research on COVID-19; presenting the intellectual content and structure visually. Further, the results systematize the relationships between these concepts and the relative volume of studies in each research area. Such results may orient researchers to what extent concepts are integrated/closely linked – which may guide and inspire them to identify and address research gaps and apply insights across research traditions. Further, it may guide researchers to sets of search terms that will yield more relevant results. The second is a network graph of published articles, where they are organized by similarity, calculated by comparing reference lists. The interactive format of the output allows the reader to search for a given article and identify related ones, and then simply click on the related article and be directed to the full text online. This method of identifying related literature complements the more common literature search, based on terms, as similarity is based on reference lists, and thus opens for identifying novel and valuable connections between research articles. Our suggested approach will give a wide range of readers—researchers, health care personnel, policy makers, funding bodies, journalists, or interested members of the general public—a complete overview of the COVID-19 research field that it would otherwise be difficult to attain. The most important contribution is that the analyses on the webpage are dynamic and show what the structure of the field looks like as more research emerges and the field develops.

## Methods

Bibliometric analyses are the quantitative evaluation of a corpus of scientific work, based on bibliographic and article metadata. In this study we apply keyword co-occurrence and bibliometric coupling, in order to identify the structure research on the COVID-19 pandemic. The results are subsequently mapped visually [4]. Science mapping based on bibliometric analysis is composed of several steps, literature search, cleaning and analysing the bibliographic data, before the results are visualized and and interpreted [3]. We further apply social network analysis to analyse the bibliometric network graphs. The literature included in this study is collected from various sources. There are two reasons for the decision to use a single database for each analysis rather than combine results from different sources. First, few databases have the required bibliographic data needed for the analysis, second the errors created when converting between formats are not likely to merit the added value. The network and bibliometric analysis are thus run on a sample, rather than all articles. As this sample represents a very large proportion of all articles, the results are considered valid [5].

For the different analysis we choose the databases deemed to have the best bibliometric data available for our unit of analysis. For the co-occurrence analysis, we use data collected from PubMed, as it is the most comprehensive database for COVID-19 related literature, and is updated daily [6]. This was confirmed after conducting the search in both Scopus, PubMed and ISI Web of Science. As PubMed does not include reference lists, we use data collected from Scopus for the bibliometric coupling analysis. Scopus was chosen over ISI Web of Science because of more frequent updates and focus on life sciences [6].

The search criteria include the following terms: “COVID-19”, “SARS-CoV-2”, “severe acute respiratory syndrome coronavirus 2”, “2019-nCoV” and “2019 novel coronavirus”. As the spread of COVID-19 was first detected in December 2019, in Wuhan, China, research included in this study is from 2020 and onwards. In Scopus we searched for these terms in TITLE-ABS-KEY, and in PubMed in Title/Abstract (full search terms for each database are provided on the website). To avoid confusion with research on other strains of the Corona virus, this term is not included. The analysis was conducted using the VOSviewer 1.6.14 software [7], which is generally accepted to represent best practice in the science mapping literature [8]. The data is cleaned in that singular and plural forms of a term are combined as are synonyms. The search terms are excluded from the analysis, as are generic terms, like the word “study”. The file showing all cleaning is provided on the website.

### Analytical strategy

#### Keyword co-occurrence analysis

The conceptual idea behind keyword co-occurrence analysis [9] is that when a set of words occur in different documents, the concepts behind these words are likely closely related. By algorithmically extracting keywords and quantitatively analyzing the content of a group of documents we can establish how closely related they are. From these results, we can build a conceptual network structure of the research field [10]. In our corpus from PubMed, this includes both author-generated keywords and MeSH terms.

We used the keywords to construct a two-dimensional keyword-map, where the layout is based on a framework for mapping and clustering, in the VOSviewer software [7]. Keywords were mapped so that keyword relatedness is associated with proximity on the map. The size of the nodes reflects keyword frequency, and the weight of connecting lines indicates in how many articles the keywords co-occur. The keywords are clustered using an approach akin to modularity-based clustering [7]: all keywords are analysed and placed in a cluster where they co-occur most frequently, signified by colour. The naming of the identified clusters aimed at reflecting its most prevalent themes, using the coding principles of grounded theory [11], including the steps of open and axial coding, in order to identify common topics in the clusters. The process included conducting a cluster analysis with higher resolution of the co-occurrence map, and the application of weighted degree centrality [12] to identify the most prominent terms. The network map gives an overview of the research field, also showing which topics are studied in conjunction to each other, and it can give an indication of there may be knowledge gaps in the research field.

#### Bibliometric coupling analysis

With the bibliometric coupling analysis we examine documents reference lists to identify shared references. The extent of overlap between reference lists is a measure of the strength of connection between documents [13]. A large overlap, when two documents share many references indicate a probability that the documents are on a related topic. Where there is little overlap, it suggests the documents are based on distinct literatures and likely cover different topics. We present a two-dimensional map, created using VOSviewer, where the layout is determined using a unified framework for clustering and mapping [14]. The articles are located so that the distance between the nodes represent their relatedness and are grouped in clusters, which indicates a shared theme. The size of the node indicates the number and strength of connections to other articles. The articles that do not have a reference list, or that does not share any references with other articles, are not allocated to a cluster.

To identify important nodes in each network graph, we calculate two centrality measures. Weighted degree centrality (referred to as centrality) which is the sum of links a given node has to other nodes, taking the strength of the link into account. This measure indicates the importance of a node. In the co-occurrence network graph some keywords connect the whole or large parts of the network and represent generally important terms, not specific to any one topic. We identify these as having a high bridging centrality, a metric for how often a node is on the shortest path between any other two nodes [15].

## Results

The results section is intentionally kept brief, as results will evolve as more research becomes available and will be reported to greater extent on the associated website: https://covid19biblio.com/ The results presented in this study are as of March 23rd, 2020.

### Keyword co-occurrence analysis

**Fig. 1:**
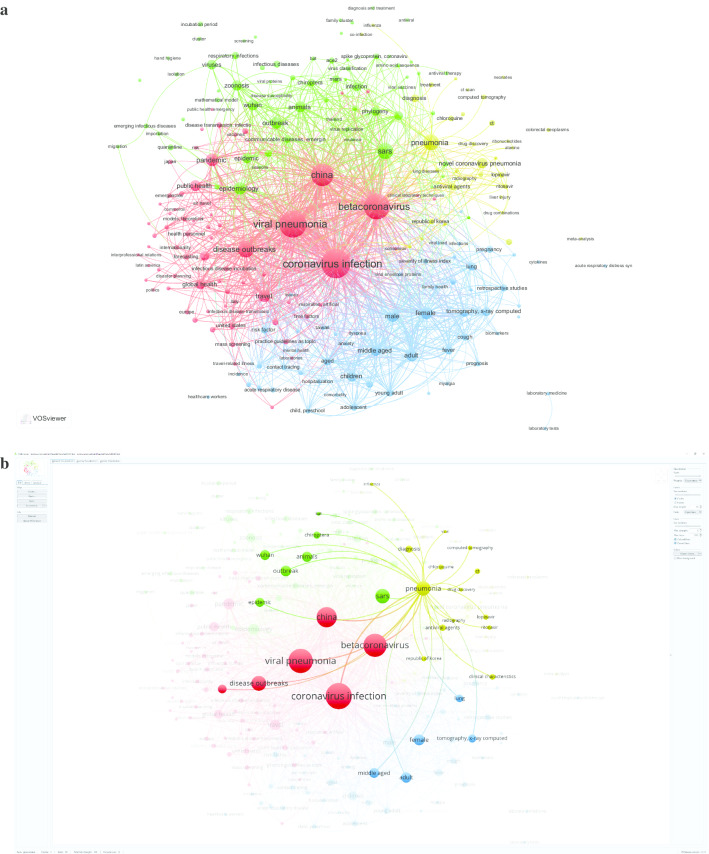
**a** Keyword co-occurrence network graph of COVID-19 research topics. Red cluster: Health and pandemic management; Green cluster: The disease and its pathophysiology; Blue cluster: Clinical epidemiology of the disease; Yellow cluster: Treatment of the disease. Size of circle shows the relative number of occurrences of a keyword, and weight of line indicates the frequency two keywords are linked. To view all the links, you can access the interactive map using the Map and Network files found at OSF: https://osf.io/54gqw/ and the VOSviewer software. We used the VOSviewer version 1.6.14. To view keyword analysis from other time periods, please see https://covid19biblio.com/keyword-co-occurrence/. **b** A Section of (**a**), keyword co-occurrence network graph of COVID-19 research topics. Displayed in the interactive tool, where “pneumonia” is selected to show other major keywords it is researched together with. The figure also shows that in addition to linking with keywords within its own cluster, the keyword “pneumonia” links with topics in the other clusters

There were 225 keywords that occurred in three or more manuscripts. These divided into four clusters. These are shown in the keyword co-occurrence network diagram (Fig. 1a). While the contents of the clusters are likely to develop in future analysis the current clusters seem to represent the following (see Fig. 1a for colour reference):

Cluster 1 (red) relates to “Health and pandemic management”, and include topics like the pandemic, the impact on global health, the disease outbreak, infection control and travel. Within this cluster, we found the topic of “Global health politics”, which in addition to terms on the pandemic and the impact on global health included terms like public health, disease transmission and health planning. Further, within this cluster, we found the topic of “Pandemic politics”, which in addition to the terms disease outbreak and infection control included terms like mass screening and health personnel.

Cluster 2 (green) related to “The disease and its pathophysiology” of COVID-19, including topics like its genome and its relationship with Severe Acute Respiratory Syndrome (SARS), but also topics like epidemiology and its outbreak and that it is a zoonosis. Within this cluster, we found the topic of “Viral biology”, which in addition to terms on its genome and SARS, included terms like phylogeny and disease reservoirs. Further, within this cluster, we find the topic of “Viral spread”, which in addition to the term epidemiology, includes terms like quarantine, importation and incubation period. Lastly, this cluster contained the topic “Basic clinical medicine”, which in addition to the terms outbreak and zoonosis, included terms like transmission and mortality.

Cluster 3 (blue) related to the “Clinical epidemiology of the disease”, including topics like age (aged, children, adolescent) and gender (male and female), risk factor, population surveillance and pregnancy. Only one major topic was identified within this cluster: “Clinical characteristics”. In addition to the terms on age and gender, this topic included terms like prognosis, myalgia, biomarkers and laboratory medicine.

Cluster 4 (yellow) related to “Treatment of the disease”, with terms like antiviral agents, diagnosis, ritonavir and drug combinations.

Four keywords act as bridging terms in the keyword co-occurrence network graph, connecting the clusters and graph together to a high degree. These are: China, Betacoronavirus, Viral pneumonia and Coronavirus infection.

Figure 1b illustrates a section of Fig. 1a, and shows what the graph looks like when it is displayed with the interactive tool, when you select the term “risk assessment”.

### Bibliometric coupling analysis

**Fig. 2:**
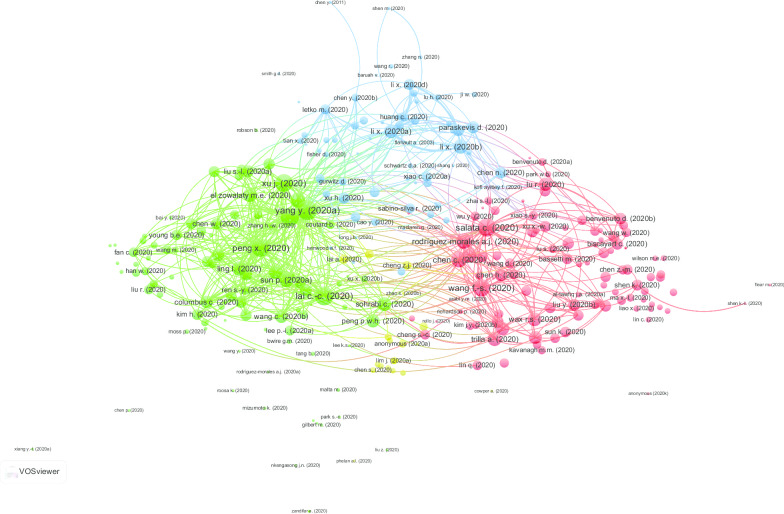
Network visualization of bibliometric coupling of COVID-19 research. Size of the circle shows the relative number of total links to other articles, the proximity between circles indicate similarity, as gauged by how many references articles share. The weight of the line indicates the number of shared references (set minimum to 4 for clarity). To examine how individual articles link to other articles, and adjust the resolution, you can access the interactive map using the Map and Network files of interest found at OSF: https://osf.io/54gqw/ and the VOSviewer software. We used the VOSviewer version 1.6.14. For coupling analyses of COVID-19 literature from other time periods, please see https://covid19biblio.com/coupling-analysis/

Of the 411 articles available in Scopus, 280 of them included a reference list and were included in the bibliometric coupling analysis. The network graph subsequently comprised of four clusters, as shown in Fig. 2.

Cluster 1 (red) related to contributions giving an “Overview of the new virus” and included literature on a wide variety of topics like genomic characterization, pathology, diagnosis and treatment, recognition of this pandemic stemming from China, and first lessons from clinical management. The subgroup “Commentaries on the new virus” related to literature on the first cases, the infection being without borders, therapeutics and triage. Another subgroup, “Commentaries on the phenomenon”, included literature on the virus spread and the reproductively of the virus.

Cluster 2 (green) related to contributions to “Clinical medicine”, including topics such as transmission routes, epidemiology, clinical characteristics of patients (age, gender, fever, abdominal symptoms). One subgroup related to “Clinical medical characteristics”, with topics like those mentioned above. Another subgroup consisted of literature on “Endemic areas” such as China, Wuhan, Korea, Thailand and Africa. A smaller subgroup, “Comparisons with SARS”, consisted of literature comparing the current pandemic with SARS.

Cluster 3 (blue) “On the virus” consisted of literature on topics ranging from the origin of the virus, its genome, possible therapeutics and early research on these topics. A major subgroup in this cluster, “the virus’ origin and its genome”, included topics like zoonotic spillover and transmission, but also early literature on the outbreak of the pandemic. Another subgroup, “Possible therapeutics”, included literature on chloroquine, Chinese herbal medicine, anti-viral drugs and the ACE2 receptor. A third large subgroup, “Early research on genome and therapeutics” included literature on receptors, transmission, detection and vaccine.

Cluster 4 (yellow), was by far the smallest cluster. It consisted of literature on “Reproduction rate and spread”, but also covers literature on screening, the pandemic and treatment. While this cluster was less well defined in the present analysis, this may change as the corpus of published work grows in the coming weeks and months.

Accessing this network diagram online, allows the reader to search for articles by author, identify related studies, and directly access the article by clicking on it.

## Discussion

With this paper, we introduce a dynamic resource that will give a swift overview of how the structure of the research on COVID-19 is developing. To illustrate what this can look like, we show the structure of the research as it stands a few months into the pandemic. The structure is likely to change as we progress from the early to later stages.

### Implications and discussion of stakeholders who may find the resource useful

We believe that this resource will be valuable for a wide readership, including for (1) policymakers and funding bodies; this can give an overview of whether the research being conducted is in line with the prioritized areas listed by the expert group at WHO [2]. (2) Researchers; the overview of what is being done in the entire COVID-19 research field, help them identify research gaps [2]. Further, the resource provides a quick access to and overview over existing research on their topics of interest—and how it links to other COVID-19 topics. (3) Health care personnel; some literature aims to provide guidance to health care personnel working with patients with COVID-19. The online resource will help them locate relevant literature more quickly. The usefulness of the resource is not limited to the above-mentioned groups. It may also be of use to (4) journalists: news outlets base some of their reporting on COVID-19 on research literature. The resource can be of value for journalists looking for updated information on a specific combination of COVID-19 topics for a news story. (5) Members of the general public; some may be particularly interested in a specific topic but find limited information about that topic in the news. For example, a couple expecting a baby may want more information about the risk that the virus poses to their unborn child. By searching the dynamic network graphs, they can locate relevant search terms to locate specific research on the topic.

### Methodological considerations

There are no restrictions to the literature included apart from the requirements to be indexed in the respective databases. This means that both high quality and more dubious research is given equal weight. No restrictions were made in terms of language or countries of origin regarding the literature included, however, only research indexed by PubMed and Scopus are included, in the keyword co-occurrence analysis and the scoping analysis respectively. And while both databases are extensive, they are not complete. The analyses are therefore based on a sample, rather than the complete COVID-19 literature. Third, bibliometric science mapping only provides an overview of a research area and is not a substitute for in-depth reading. Fourth, unlike other overviews of the literature, that may limit inclusion of literature to published research articles, we include all types of publications in PubMed and Scopus. By doing so, with clearly defined search and analytical criteria, we increase the transparency and replicability of the process and reduces potential researcher bias in the selection of studies included in the study.

We do this as during a time of crisis where relevant research must be made available as soon as possible, research may be published in a variety of formats in the interest of expediency.

## Conclusion

To meet the challenges of the pandemic, we wish to harness the power of collaboration, and therefore invite other researchers and research institutions who wish to contribute to making this an even more valuable resource, to contact us. There is scope to expand this dynamic resource, in terms of depth and additional analysis. There are several additional bibliometric and network-based analyses that can be conducted to examine the evolving COVID-19 literature, some of which can be reported online at the associated website: Covid19Biblio.com. For example, comparative analysis of evolving research across periods, author/institution collaboration analysis showing who is working on various topics, and comparative analysis with past epidemics. This is a time to realize good ideas and bring them together.

## Data Availability

Raw data can be accessed by using the search terms provided in the methods section and repeating the search in PubMed and/or Scopus. In addition, all the files needed to access the network graph files can be accessed through the project folder at the open science framework (OSF) site: https://osf.io/54gqw/. These files can be opened in the freely available VOSviewer software. Analyses from different periods during this pandemic, and information about how to use the data is available on the website that is an extension of this article: https://covid19biblio.com/

## Abbreviations

COVID-19: Coronavirus disease
OSF: Open science framework
SARS: Severe acute respiratory syndrome
SARS-CoV-2: Severe acute respiratory syndrome coronavirus 2
WHO: World Health Organization
2019-nCoV: 2019 Novel coronavirus
